# Implementation of an ED surge management platform: a study protocol

**DOI:** 10.1186/s43058-021-00247-1

**Published:** 2022-03-02

**Authors:** Nahid Rahimipour Anaraki, Jennifer Jewer, Oliver Hurley, Hensley H. Mariathas, Christina Young, Paul Norman, Christopher Patey, Brenda Wilson, Holly Etchegary, Dorothy Senior, Shabnam Asghari

**Affiliations:** 1 Centre for Rural Health Studies, Faculty of Medicine, Memorial University of Newfoundland, St. John’s, NL A1B 3V6 Canada; 2 Faculty of Business Administration, Memorial University of Newfoundland, St. John’s, NL A1B 3V6 Canada; 3 Eastern Health, Carbonear Institute for Rural Research and Innovation by the Sea, Carbonear General Hospital, Carbonear, NL A1Y 1A4 Canada; 4 Discipline of Family Medicine, Faculty of Medicine, Memorial University of Newfoundland, St. John’s, NL A1B 3V6 Canada; 5 Community Health and Humanities, Faculty of Medicine, Memorial University of Newfoundland, St. John’s, NL A1B 3V6 Canada; 6 Faculty of Medicine, Memorial University of Newfoundland, St. John’s, NL A1B 3V6 Canada; 7 Patient Advisory Council, NLSUPPORT, Memorial University of Newfoundland, St. John’s, NL A1B 3V6 Canada

**Keywords:** SurgeCon, Emergency department, Implementation science

## Abstract

**Background:**

Emergency departments (EDs) around the world are struggling with long wait times and overcrowding. To address these issues, a quality improvement program called SurgeCon was created to improve ED efficiency and patient satisfaction. This paper presents a framework for managing and evaluating the implementation of an ED surge management platform. Our framework builds on the Reach, Effectiveness, Adoption, Implementation, and Maintenance (RE-AIM) framework to structure our approach and the Consolidated Framework for Implementation Research (CFIR) to guide our choice of outcome variables and scalability.

**Methods:**

Four hospital EDs will receive the SurgeCon quality improvement intervention. Using a stepped wedge cluster design, each ED will be randomized to one of four start dates. Data will be collected before, during, and after the implementation of the intervention. RE-AIM will be used to guide the assessment of SurgeCon, and guided by CFIR, we will measure ED key performance indicators (KPI), patient-reported outcomes, and implementation outcomes related to SurgeCon’s scalability, adaptability, sustainability, and overall costs. Participants in this study consist of patients who visit any of the four selected EDs during the study period, providers/staff, and health system managers. A mixed-methods approach will be utilized to evaluate implementation outcomes.

**Discussion:**

This study will provide important insight into the implementation and evaluation techniques to enhance uptake and benefits associated with an ED surge-management platform. The proposed framework bridges research and practice by involving researchers, practitioners, and patients in the implementation and evaluation process, to produce an actionable framework that others can follow. We anticipate that the implementation approach would be generalizable to program implementations in other EDs.

**Trial registration:**

• Name of the registry: ClinicalTrials.gov

• Trial registration number: NCT04789902

• Date of registration: 03/10/2021

Contributions to the literature

- This study presents an actionable, comprehensive guideline for the implementation of interventions within ED settings.
- The intervention builds on a combination of RE-AIM to structure the approach and CFIR to guide the choice of outcome variables.
- This study adds to the literature by using qualitative as well as quantitative strategies to comprehensively address all RE-AIM dimensions.
- This study involves a multi-disciplinary planning and implementation team who play significant roles in implementing and evaluating the intervention program.
- Patient engagement is fundamental to this research project. Patients are provided with a variety of opportunities to engage in different stages of the intervention.

## Background

### SurgeCon program

Emergency departments (EDs) around the world are struggling with long wait times and overcrowding [1–3]. Such wait times are an issue as they intensify the chance of patients leaving without being seen by an ED doctor [4, 5]. Canada has one of the longest average ED wait times compared to peer-industrialized countries with steadily increasing median wait times from 2.8 h in 2017–2018, 3.2 h in 2018–2019, to 3.3 h in 2019–2020 [1, 6]. In Newfoundland and Labrador (NL), Canada, excessively long ED wait times have made the province a prime example of issues EDs are facing in the country [7–9]. To address this issue, our team is implementing and will evaluate a quality improvement initiative called SurgeCon, which includes a protocol-driven software platform, to decrease wait times and enhance the sustainability of NL’s health system without crucial workforce modifications. SurgeCon was developed to allow frontline healthcare providers to predict and alleviate surges in patient volume based on set of tactical steps and decision-making tools. We have chosen a stepped wedge randomized control trial design to evaluate the effects of SurgeCon on ED operations.

### Rationale for the implementation and evaluation framework

It is challenging to implement and evaluate complex interventions, such as SurgeCon, in a hospital setting. Hospitals have a unique context, process, system, and population, and hence they experience unique challenges. It is well-documented that many health services interventions fail to be fully implemented, produce effective changes, or be sustained for long-term. This is especially true when the innovation requires complex alterations in clinical practices such as improved communication and cooperation among clinicians, researchers, and administrators or modifications in the organization of care [10–13]. These complex interventions demand comprehensive evaluations of process and the impact created by the intervention to capture not only information related to its implementation but also its effects on organizations and individuals in the long run [14]. Therefore, it is important to develop a framework to guide such implementation and evaluation. Following best practices [13], the framework suggested in this study was developed based on the accumulated evidence and experiences at a pilot site, Carbonear Hospital, NL [15, 16]. We are also guided by RE-AIM and CFIR [17, 18]. We selected these frameworks because they enable us to consider the context which includes the needs of the population, the work practices, the culture, and the system as a single entity during implementation. We recognized that the intervention is not just the system; it also includes the other steps that are part of the implementation (training, documenting work practices/processes, etc.). We made a conscious effort to include these in our framework.

RE-AIM was developed by Glasgow et al and has been used in over 400 studies as a means of improving the external validity of measures and the implementation of interventions in healthcare [17, 19, 20]. RE-AIM highlights not only the representativeness of participants but also the setting, as both have a critical role in public health interventions [17]. RE-AIM consists of five dimensions reach, effectiveness, adoption, implementation, and maintenance, which have been used to guide the implementation of health care interventions. While RE-AIM provides researchers with a comprehensive plan for evaluating an intervention, CFIR, which is composed of five domains (intervention characteristics, outer setting, inner setting, characteristics of the individuals, and the process of implementation) discloses the “black box of the ‘I’ (implementation) component” ([18], p.12). CFIR has been utilized to explain and identify barriers and facilitators to adoption, implementation, and maintenance. These frameworks complement each other and have been applied successfully together to “develop testable, theory-informed implementation strategies” ([21], p. 9). To our knowledge, this paper presents one of the first comprehensive implementation and evaluation guidelines designed for the ED which uses both the RE-AIM and CFIR frameworks.

## Methods

### Aims and objectives

The primary aim of this paper is to identify and describe the context in which implementation occurs and the factors that influence implementation in a hospital setting [22, 23]. Based on the literature and our pilot assessment of SurgeCon, we have included a multi-faceted implementation strategy to help support the successful uptake of SurgeCon. Our secondary implementation aim is to describe the use of these strategies in different ED contexts and identify any changes that can be made to improve the uptake and sustained use of SurgeCon.

### Study design

The intervention will be implemented in the four hospitals using a stepped-wedge cluster randomized trial during 31 months [24]. The study will begin with a 6-month control period. Throughout this period, all patients who visited 1 of the 4 selected EDs will receive usual care. Then, each of the four hospitals will be randomly selected at a 6 month interval to initiate the SurgeCon intervention at that site. Randomly selected hospitals will undertake training and implementation of the intervention for 1 month and will remain in an intervention state until the end of the study period. By the end of the study, all four hospitals will operate using the SurgeCon ED management platform.

### Study setting

EDs with 24/7 on-site physician support in the Eastern Health (EH) region of NL were selected in this clinical trial: (1) Health Sciences Centre (Urban/Tertiary), (2) St. Clare’s Mercy Hospital (Urban/Tertiary), (3) Dr. G.B. Cross Memorial Hospital (Rural/Secondary), and (4) Burin Peninsula Health Care Centre (Rural/Secondary).^1^ Participants of this study consist of patients who visit any of the four selected EDs during the study period, providers/staff, and health system managers.

### Intervention

The SurgeCon quality improvement program is an intervention with the aim of improving wait times, patient satisfaction, provider satisfaction, and value of care in the ED. The three components of the intervention include (1) restructuring ED organization and workflow, (2) implementing an action-based surge capacity plan, and (3) fostering a patient-centric environment. The purpose of the restructuring of department workflow and organization is to promote communication across provider types (e.g., physicians, mid-level providers), offer an alternative method for stable patient priority setting that improves department efficiency while maintaining patient safety, and provide strategies to reduce door-to-doctor time (e.g., the time between arrival and first contact with a doctor). SurgeCon also includes an eHealth platform of an action-based surge capacity plan to help frontline staff and administrators manage the level of demand in the ED by actively tracking the number of patients in the department, the available resources, and the level of care required for each patient. Frontline staff report information through a data entry portal, which are processed by an algorithm. The algorithm determines which staff to notify and assigns specific actions to maintain a constant flow of patients. The SurgeCon intervention also aims to establish a more patient-centric environment for patients by improving the physical environment of the department (e.g., chairs in waiting room, reduce clutter in assessment spaces, and improve appearance of rooms) and wayfinding signage, to assist patients with navigating the department and hospital. To help support the implementation of the intervention, the majority of frontline staff at selected sites will be required to complete a SurgeCon training course. The course provides content for frontline staff to become more familiar with SurgeCon’s alternative management framework, strategies for managing patient flow, and the principles of patient-centered care.

### Implementation team composition

The planning and implementation team for this study is multi-disciplinary, including qualitative and quantitative researchers, clinical trial experts, implementation scientists, healthcare economists, health informatics researchers, healthcare professionals, health system managers, frontline healthcare staff, and patients, all of whom play significant roles as patient research partners, facilitators, site coordinators, champions, and members of various working groups and committees (i.e., Implementation, Innovative Clinical Trial, Patient Engagement, Steering, Executive) in implementing and evaluating the intervention program (see Table 1).

**Table 1:** Implementation personnel list

	Implementation personnel	Explanation
1	SurgeCon facilitator	The facilitator is a member of the Implementation Working Group who was involved in the development of SurgeCon in Carbonear. The facilitator will be designated to be one of the main points of contact for local teams during the implementation process
2	Site coordinator	Site coordinators are nurses who will be performing on-site research-related tasks and assisting with the interview recruitment
3	Frontline SurgeCon champions	Once the site assessment is complete, ED management will be asked to select a member of the local ED frontline team who will receive additional training either in-person or remotely. That individual will then be an ongoing point of contact for ED staff at their site who have any questions related to SurgeCon. They will also liaise with the research team at regular intervals to discuss any practical or technical issues with using SurgeCon. Also, observation will be utilized by champions to collect information during the readiness assessment period to ongoing implementation evaluation
4	Implementation working group	The SurgeCon implementation working group is responsible for overseeing and guiding the implementation of the intervention at each of the selected sites
5	Innovative Clinical Trial (iCT) working group	The iCT working group supports the team with their expertise in methodology and ensures the validity and precision of the study
6	Patient engagement working group	The patient engagement working group will oversee and guide patient engagement and patient-oriented research in all sites.
7	Executive committee	The executive committee (all steering committee members, plus the patient advisor, payer representative, key strategic area leaders, and a policymaker) will oversee the whole project and have the authority to determine priorities and supervise the general course of operations
8	Steering committee	The steering committee (Lead researcher, clinical lead SurgeCon facilitator, research manager) will manage daily operations to ensure the project adheres to the Rewarding Success agreement and that the highest standards of scientific rigor are maintained
9	Patient research partners	Patient research partners are patients who are also members of the research team. Patient research partners provide their perspective and help guide decisions to ensure the research produces outcomes and knowledge that can be used to help address the needs and priorities of the local populations they represent
10	ED team	The ED team includes all physicians, nurses, allied health, and other personnel who work at the four selected EDs. They will participate through interviews and report questions, concerns, and issues to site coordinators and champions

We involve stakeholder groups (e.g., patients, decision makers, researchers, clinicians, etc.) in all stages of SurgeCon’s implementation. Collaboration is not limited to the providers and managers, as the role of patients is crucial. A contribution of this study is the guidance we provide to researchers about how to engage patients in a systematic way to redefine and reassess intervention programs to make them more culturally and contextually relevant. To shape our patient engagement strategy, we used four leading principles [25] such as (1) patient initiation (allowing patients to participate in the research process), (2) building reciprocal relationships (all individuals being treated as equal partners), (3) co-learning (researchers and patients learning from each other), and (4) re-assessment and feedback (routinely consulting with patients and making improvements accordingly). Our structured framework provides patients with a number of different opportunities to be involved in the implementation and evaluation process, ranging from commitments that require less involvement (e.g., surveys, interviews, etc.) to full, ongoing participation through team membership.

### Data collection

Implementation outcomes will be collected through a mixed-methods approach, including (1) semi-structured, in-depth interviews with patients and ED staff, (2) observation by researchers and champions, (3) survey instruments, (4) wait times data, and (5) data from SurgeCon’s dashboard system and evaluated by patients, providers/staff, and health system managers.

Semi-structured, in-depth interviews (conducted via phone, digitally recorded, and lasting 45 to 60 min) and observation will be utilized to collect information during the exploration period to ongoing implementation evaluation (e.g., assessing ED’s physical layout, barriers, and enablers to SurgeCon adoption and sustained use, and examining ED contextual constructs and factors).

In the first stages of qualitative data collection, purposeful sampling will be applied to maximize the chance of obtaining rich data for central research topics from “information-rich cases” which will subsequently be followed by theoretical sampling [26]. Theoretical sampling occurs when the analyst simultaneously collects and analyzes data to determine where to collect additional data for the purposes of theory development [27]. This strategy involves sampling that is not predefined prior to the commencement of the study and instead emerges through data collection and analysis [28].

Individuals selected to be site coordinators will be responsible for assisting with the scheduling and coordination of interviews with ED staff. Staff members who indicate that they do not want to participate in the study will not be contacted. The site coordinator will facilitate the pre-interview process for staff members who have expressed interest in completing the interview (e.g., consent form, selecting a private location for the interview to be conducted) or will provide contact information of staff members to the research team. The research team member will then complete a web-conference/telephone interview with the participant in a private setting. Recruitment will continue until data saturation  is achieved.

Quantitative data will be collected from several sources. Patient satisfaction and patient-reported experience with ED wait times are collected using a survey questionnaire. Randomly selected individuals who agreed to complete the survey will be contacted within 3 to 5 days of ED discharge during the trial period. We will collect factors on patients as well as services that influence patient satisfaction before, during, and after intervention implementation.

The research team will also be receiving SurgeCon dashboard aggregate data from MOBIA, a company responsible for the development and maintenance of the eHealth platform and the data captured through the SurgeCon data entry portal. Currently, dashboard data is manually entered by a charge nurse through a data entry portal. The data is routinely collected and is recommended to be entered at a minimum interval of 2 h. The data entered will vary based on the variables selected by the department frontline staff and management. Variables include but are not limited to number of beds available within the department or inpatient units, number of patients in waiting room or left to triage, number of admissions in ED, and ambulances not off loaded, among other options. Data captured through SurgeCon’s dashboard system is then processed via an algorithm that weighs and scores each variable to help determine the availability of resources and the level of demand in the department. The overall score calculated by the algorithm will determine which recommended actions will be given to the frontline team and who will be automatically notified to mobilize resources and support to assist with emergency department operations and maintain patient flow.

Lastly, health administrative data will be provided by the Newfoundland and Labrador Centre for Health Information (NLCHI) who is the data custodian for most provincial health databases. Our data request includes data related to patient record information for those who visited an ED during the specified time period, department finances, patient demography, mortality, physician claims (fee for service), hospitalizations, diagnostic services, and pharmacy among other variables. We also requested aggregate emergency department performance data which includes key performance indicator (KPI) data such as physician initial assessment, length of stay, number of patients left without being seen, patient volume, hospital occupancy rate, and readmission rate.

In order to detect a change in patient ED wait times, we estimated a sample size of *N* = 20,280 or 169 patients per month per hospital for 30 months for 4 hospitals. The sample size for ED wait times has the potential to detect a 15% change in wait times. To detect a change in patient satisfaction, we estimated a sample size of 1320 for the patient survey or 11 patients per month per hospital for 30 months for 4 hospitals. The sample size calculated for patient satisfaction has the potential to detect a 30% change in patient-reported experience and satisfaction (these calculations assume 5% type I error, 80% power, and an intra-cluster correlation (ICC) of 0.1).

### Data analysis

Exploring a complex social context—i.e., ED—which often experiences unique challenges at both individual and organizational levels can benefit from inductive approaches to comprehensively interpret the complexity. Qualitative data from in-depth and semi-structured interviews and observations will be analyzed based on Straussian Grounded Theory (GT). The inductive logic of GT seeks to fully capture the world of participants by focusing on their perceptions, intentions, views, actions, understandings, and experiences associated with the phenomena rather than purely concentrating on theories [29]. The main task of the analyst is to create a set of evolving categories and define their properties which are integrated into a theory [27]. To achieve this goal, the researcher begins with open coding. During the open coding process, the first stage of data analysis is to break down collected data into concepts, their dimensions, thoughts, and ideas. This provides an opportunity for the researcher to find similarities and differences and to categorize similar occurrences and behaviors into the same group. Data generated during the open coding process that resemble one another are subdivided into different codes. This subdividing of data assists in the development of a comprehensive explanation of the phenomenon, which is the purpose of axial coding, the second stage of the coding process. The extraction of a core category from this initial two-stage process is the task of the investigator during selective coding. All stages in the coding process will be conducted by a qualitative researcher. Codes and categories will then be reviewed by members of the Implementation Working Group to reach a consensus. At the end of the study, the credibility of results will be enhanced by member checking, data triangulation, and peer debriefing.

We plan to present descriptive information related to the characteristics of each hospital and the patients that visit them. Data collected through telephone interviews with patients will allow for the comparison of patient satisfaction and experience measures pre and post intervention. Healthcare staff at each of the four sites will also be surveyed to determine their level of workplace satisfaction and to gauge the level and duration of intervention protocol adherence. If staff members from each of the four departments are found to disregard intervention protocols and guidelines, reasons for non-compliance will be collected and analyzed to help improve intervention adoption and long-term usage. We will document and report any event where the intervention produced unintended outcomes or was found to contribute to the development of a serious adverse event. The outcomes targeted by this study will be regularly reported at the end of each period and will include information such as effect size and precision.

ED wait time data, patient-reported experiences with ED wait times and patient satisfaction from pre and post intervention periods will be compared to assess the intervention’s effectiveness at producing improved outcomes using generalized linear mixed modeling (GLMM).

Hospitals will be analyzed according to their random assignment order irrespective of whether they achieved the outcome at the desired time. An intention to treat approach will be used alongside sensitivity analysis to determine whether any differences exist between those who received the intervention according to randomization, and those who were compliant and noncompliant. The data collected from the four sites during the 30-month study period will be analyzed in a timely manner to identify what areas of the intervention need to be modified in order to produce better outcomes or improve intervention adherence. All statistical data analysis will be conducted using R Version 4.1.0 in a secure server environment. Additional details on quantitative data collection and analysis are available via other works published by our team [30].

### COVID-19 impact on research operations

Due to the pandemic, staff training and research team meetings will be delivered or carried out through web-conferencing/a virtual platform. Additionally, some of the data collection was originally intended to be carried out by the research team; however, we changed the plan by involving hospital employees. For instance, hospital employees will utilize observation to collect information during the ongoing implementation evaluation. Also, the coordination of research operations at each of the sites will be completed by EH staff since the intervention sites are restricted to EH personnel. EH employees responsible for research related tasks will be jointly supervised by the research team and EH managers. Additionally, the SurgeCon platform and research instruments will be adjusted to capture and report COVID-19 data.

## Implementation and evaluation

### ED implementation and evaluation framework

SurgeCon’s implementation plan include an iterative improvement process that is divided into four stages: (1) exploration, (2) adoption, (3) active implementation, and (4) sustainment [31]. For each stage, we applied the RE-AIM framework and CFIR domains to identify the criteria that applied to our context. Table 2 provides details on the evaluation and timelines according to the implementation stages. All measures, including how each domain is mapped in the RE-AIM framework, are provided. We have expanded RE-AIM’s implementation outcomes to include outcomes recommended by CFIR [32].

**Table 2:** Implementation frameworks used to evaluate implementation outcomes

Dimensions/Variables Description	Implementation Stage (time period)			
	Exploration months 1–10 at all hospitals	Adoption 1 month at each hospital (months 11–12, 17–18, 23–24, 29–30)	Implementation Evaluation/iCT months 13–31	Sustainment months 17–48
**Reach (RE-AIM Framework)**				
Exclusions: The percentage of eligible ED sites that are excluded pre-randomization.	*√*	*√*	*√*	
Participation rate: The number of EDs that participate divided by all EDs that meet the eligibility criteria.		*√*	*√*	
Characteristics of the participant sites and non- participants sites: Assessment of the following variables: the average number of patients, the average number of staff, staff mix, staff characteristics (age, sex, years of practice) and patient characteristics (age, sex, CTAS score). This will also include assessment of potential moderating factors such as organization readiness for change.	*√*	*√*	*√*	*√*
Understand barriers and enablers to reach	*√*	*√*	*√*	*√*
**Effectiveness (RE-AIM Framework)**				
Health system level: Length of Stay, Time Until Physician Initial Assessment, Left Without Being Seen			*√*	
Patient-reported level: Satisfaction, patient reported experiences of ED service			*√*	
Understand barriers and enablers to effectiveness			*√*	
**Adoption (RE-AIM framework)**				
The proportion of health care providers who engage in SurgeCon activities among those who agreed to participate in the study (acceptability, appropriateness)	*√*	*√*	*√*	
Understand barriers and enablers to adoption	*√*	*√*	*√*	
**Implementation (RE-AIM framework)**				
Fidelity of staff training			*√*	
Fidelity of intervention delivery			*√*	
Adaptations			*√*	
Implementation cost				*√*
Understand barriers and enablers to implementation				*√*
**Maintenance (RE-AIM framework)**				
Institutionalization: long-term adoption of SurgeCon				*√*
Cost of maintaining the intervention				*√*
Understand barriers and enablers to maintenance			*√*	*√*
**Scalability (CFIR framework)**				
Intervention Characteristics: stakeholders’ perception, complexity of intervention		*√*	*√*	
Outer Setting: health system policy, patients’ needs	*√*	*√*	*√*	*√*
Inner Setting: resources, leadership	*√*	*√*	*√*	*√*
Characteristics of Individuals Involved: knowledge, attitude	*√*	*√*	*√*	*√*
Process of Implementation: planning and training		*√*	*√*	

#### Stage 1: Exploration (site assessment)

The working group will complete five activities during the exploration phase including delivering a virtual presentation to inform ED staff (paramedics, nurses, and physicians) and management of upcoming operational changes, observing the ED’s physical layout to make improvements necessary for implementation, recording the clinical and a demographic characteristics of EDs and patient to gather information on emergency department organization and workflow, conducting patient telephone interviews and semi-structured patient interview to capture their live experiences and feedbacks, and conducting semi-structured interviews to clarify barriers and key performance issues. The four targeted EDs vary in terms of staff composition, layout, physical footprint, appearance, and resources. Information collected through site assessments will allow the working group to customize the components of the SurgeCon platform according to the needs of each ED. Using this information, the team will identify and implement elements of the intervention that are applicable and compatible with each ED (see Table 3).

**Table 3:** Description of activities during the exploration phase

Activity	Objective	Target groups (ED, staff, patients, patient advisors)	Measurement tool and method	Outcome of interest
1. Deliver a presentation	Prepare staff for upcoming changes	ED staff	Virtual presentation	Improved understanding of SurgeCon and obtaining the highest level of support possible
2. Observing the ED’s physical layout	To gain insight into the best next steps to improve layout and gain the greatest efficiency	ED	Qualitative observational method/site assessment checklist	Improved area of operations
3. Recording the clinical and demographic characteristics of hospitals and patients	To assess ED’s organizational and workflow structures	ED	Site assessment checklist	Understanding ED characteristics
4. Conducting patient-reported experiences, patient satisfaction telephone interviews, and semi-structured patient interview	To consult with patients, get feedback from them, study their lived-experiences	ED patients	Patient-reported experiences and patient satisfaction survey and semi-structured patient interview guide	Develop strategies to overcome the challenges of patient satisfaction
5. Conducting interview about barriers and enablers	To study the organizational climate of the ED before the intervention and consult with ED staff to gauge interest in implementing a new ED management system	ED staff	Semi-structured interview	Resolve potential contextual barriers to successful implementation

#### Stage 2: Adoption (SurgeCon installation)

During the adoption stage, the working group will complete seven activities, which will be initiated by the SurgeCon initialization across the four study sites in a stepwise manner to help ED staff (paramedics, nurses and physicians) manage their actions to actively reduce patient surges and wait times and increase patients’ access to emergency medical care. To test SurgeCon’s digital whiteboard application, we will validate information extracted from the platform’s data repository and analyze end user feedback to help inform iterative software updates. SurgeCon staff training will occur during this stage, while additional training and support will be made available to all sites throughout the study period. The installation of hardware and software assets for the intervention’s e-Health component as well as training will be carried out during adoption phase. We also plan to post department level data in a prominent area of the ED monthly (depending on the discretion of the sites) and also circulate individual provider KPI data anonymously. This will give physicians and also individual nurse practitioners the opportunity to know their monthly physician’s initial assessment (PIA) times compared to the ED average and targets set by ED management. Providing this data may increase physician motivation to use components of SurgeCon flow education since it will demonstrate the intervention’s capacity to reduce their door-to-doctor time—a key metric for health standards of care. At this stage, the SurgeCon facilitator who will assist local ED teams with the development of clear roles, goals, and communication strategies. The Facilitator will review SurgeCon process improvement activities, usage of the whiteboard application, and adherence to the protocol. Local champions who are leading internal change initiatives along with members of the Implementation Working Group will help prepare EDs for the Active Implementation stage of the study. Improving the overall appearance of physical spaces in the ED (e.g., waiting room, fast-track zone, examination rooms, treatment space, etc.) to improve patient satisfaction is another goal in this stage. In consultation with our local patient partners, we will renovate, redecorate, and declutter ED spaces (see Table 4).

**Table 4:** Description of activities during the adoption phase

Activity	Objective	Target (ED, staff, patients, patient advisors)	Measurement tool and method	Outcome of interest
1. Installing SurgeCon’s digital whiteboard application	To advise when to use volume-based staffing (shifting staff between areas of the hospital based on workload), appropriate and timely involvement of hospital management, and overcapacity protocols, which may otherwise be overlooked by distracted frontline ED staff	ED	Protocol-driven software platform	Online illustration of ED organization and wait time
2. Data collection	To enhance the platform and update ED staff	ED	SurgeCon platform and ED secondary data	Enhancing ED functioning
3. Training frontline SurgeCon champions	To provide ongoing learning related to using the new system and the successful uptake and adoption of SurgeCon	ED staff	Participant observation	Solving practical or technical issues with using SurgeCon
4. Staff training	To encourage ED staff to become active participants in the improvement process	ED staff	Interactive simulation course^a^, SurgeCon eHealth platform training^b^, and patient centeredness training^c^	Facilitating the implementation of quality improvement
5. Site assessment	To determine whether the site is capable of implementing the platform as intended given the level of resources and staff commitment	ED staff	Champion observation and site coordinator reports	Assisting ED staff in development of SurgeCon
6. Establishing a patient-centric ED environment	To improve the overall appearance of physical spaces in the ED	ED	Patient advisors’ observation and site assessment checklist	To improve patient satisfaction
7. Conducting patient-reported experiences, patient satisfaction telephone interviews, and semi-structured patient interview	To consult with patients, get feedback from them, study their lived-experiences	ED patients	Patient-reported experiences and patient Satisfaction survey and semi-structured patient interview guide	Develop strategies to overcome the challenges of patient satisfaction

^a^Interactive Simulation Course. This module focuses on an ED improvement flow sustainability strategy. The aim will be to provide information on the rationale underpinning SurgeCon and how it works in the practice setting using case-based scenarios. It will be interactive allowing for questions and answers and ensuring the learning outcomes are achieved

^b^This module will help ED staff: (1) become familiar with the digital whiteboard application, (2) learn how the system collects and reports information, (3) learn how to interpret system generated notices and warnings, (4) assign protocol tasks, and (5) learn effective response strategies to system notices and warnings

^c^This module includes an educational session which reinforces the following topics: (1) providing quality ED care to all patients regardless of urgency, (2) treating all patients with respect, and (3) demonstrating that the patient’s visit to an ED must always be considered necessary as they may have no other option

#### Stage 3: Active implementation (SurgeCon monitoring and evaluation)

During the implementation phase, five activities will be completed to evaluate the intervention and to determine whether implementation efforts made are sufficient to overcome barriers to a successful implementation of the intervention and produce improved outcomes. This includes fidelity of intervention (i.e., the degree to which the intervention was implemented as intended) and fidelity of training. Furthermore, we will examine contextual constructs and factors influence the intervention. For instance, mediators (factors that increase the intervention’s effectiveness in terms of producing positive outcomes), moderators (factors that influence the degree of change in targeted outcomes), and underlying mechanisms that lead to sustained organizational changes at all levels of EH’s organization [33, 34]. The ultimate goal is to culturally embed SurgeCon in each ED if it can produce consistently positive results. Adaptations will also be made to the intervention by the research team during the study period. This process will identify intervention components and implementation methods that produce the best response in staff and management in terms of intervention acceptability, commitment to change, and improved performance. Local ED teams and champions will work closely with the research team to ensure all parts of the intervention are applied as intended and evaluated appropriately. We will outline the parameters of operational processes developed during the adoption stage of the implementation plan (see Table 5).

**Table 5:** Description of activities during the active implementation phase

Activity	Objective	Target (ED, staff, patients, patient advisors)	Measurement tool and method	Outcome of interest
1. Measuring fidelity of training	To determine if the staff are trained to a well-defined performance criteria	ED staff	Training evaluation survey	Trained/competent staff
2. Measuring fidelity of intervention delivery	To determine if the sites have implemented SurgeCon as intended	ED	Software platform and champion participant observation	Successful implementation
3. Examining contextual constructs and factors	To identify and describe the context in which the intervention occurs and the factors that influence implementation	ED staff	Champion observation, site coordinator report	Understanding contextual constructs and factors to develop a plan for overcoming the challenges and ensuring successful implementation
4.Conducting patient-reported experiences, patient satisfaction telephone interviews, and semi-structured patient interview	To consult with patients, get feedback from them, study their lived-experiences	ED patients	Patient-reported experiences and patient satisfaction survey and semi-structured patient interview guide	Develop strategies to overcome the challenges of patient satisfaction
5. Conducting interview about barriers and enablers	To study organizational climate of ED before, during, and after the intervention and consult with ED staff to gauge interest in newly implemented ED management system	ED staff	Semi-structured interview	Resolving potential contextual barriers to successful implementation

#### Stage 4: Sustainment (SurgeCon maintenance and sustainability)

During the sustainment phase, the extent to which SurgeCon has become institutionalized or part of routine ED practices and policies will be examined through five activities. We will measure the following: (1) institutionalization—the long-term adoption of SurgeCon including all elements of the intervention (e.g., the action-based protocol) and its implementation strategies (e.g., champions) were retained and changed from the pilot study in Carbonear—(2) feasibility—the extent to which the intervention can be carried out in other ED departments besides Carbonear—and (3) cost of maintaining the intervention (e.g., system updates, staff training, etc.) and maintaining the restructured ED and its patient-centered environment. Also, the perceived barriers and enablers of Regional Health Authorities (RHA) managers and ED staff to the set-up and use of the SurgeCon intervention will be assessed. This will include an assessment of the different procedural components (e.g., site assessment) of SurgeCon, the day-to-day use of the different elements of SurgeCon (e.g., fast track zone, interaction with the technology and software), and the education, training, champion, and feedback strategies to determine if they were perceived to be enablers to implementation. We will use the CFIR guidelines to underpin the interview guides. We will create analytic matrices to conduct a cross-case analysis for identifying patterns of barriers and enablers and develop a collaborative approach to maintain and refine the platform over time to scale up. Additionally, to improve SurgeCon’s sustainability, all participating EDs will meet quarterly by teleconference, sharing ED team success stories, and identifying barriers to implementation, as well as approaches used to mitigate negative outcomes. They will prepare a one-page summary for the SurgeCon Executive Committee which will include the following: how ED teams plan to maintain SurgeCon’s use and operation, how to improve ED staff capacity and skills required to properly use the SurgeCon platform, and how SurgeCon can be better adapted to address general and site-specific needs. Furthermore, at this stage, it is important to identify individuals in leadership roles at both the system and hospital levels who can help drive SurgeCon’s process improvement initiatives beyond this study (see Table 6).

**Table 6:** Description of activities during sustainability phase

Activity	Objective	Target (ED, staff, patients, patient advisors)	Measurement tool and method	Outcome of interest
1. Assessing scalability of SurgeCon	To identify the extent to which the intervention can be maintained and carried out in other ED departments	ED	RE-AIM scoring and CFIR instrument	Scalable SurgeCon platform
2. Measuring the cost of implementing the intervention	To measure the cost of maintaining the intervention (e.g. system updates, staff training, etc.) and maintaining the restructured ED and its patient-centered environment	ED	Economic analysis	Cost of implementation
3. Assessing feasibility of maintaining SurgeCon	To identify the issues raised and their relevance to maintaining the intervention	ED	RE-AIM scoring instrument, champion observation, and site coordinator report	Making changes or resolving challenges as a result of intervention
4. Conducting patient-reported experiences, patient satisfaction telephone interviews, and semi-structured patient interview	To consult with patients, get feedback from them, study their lived-experiences	ED patients	Patient-reported experiences and patient satisfaction survey and semi-structured patient interview guide	Develop strategies to overcome the challenges of patient satisfaction
5. Conducting interviews about barriers and enablers to institutionalization	To study organizational climate of ED after the intervention implementation	ED staff	Semi-structured interview	Resolving potential contextual barriers to successful maintenance

## Discussion

This study provides important insights into the effectiveness of implementation and evaluation techniques to enhance the uptake of an ED management program. The SurgeCon protocol will be a valuable guide for healthcare professionals and organizations that aim to design, plan, implement, and evaluate interventions within complex healthcare settings.

It is important to consider that when applying innovations and interventions that aim to produce effective, sustainable, and enduring changes through the modification and improvement of clinical practices, organization of care, and cooperation among the implementation team, various barriers are encountered (e.g., barriers at the patient, provider, departmental, and institutional levels) [35]. This is due in part to the chaotic environment and complex setting of EDs (e.g., wait times, overcrowding, various healthcare professional team members, competing priorities among the staff, budget restrictions, staff turnover, etc.) [36]. This affirms the significance of carrying out this intervention with comprehensive actionable implementation and evaluation guidelines that translate research into practice and can be followed by researchers in their future implementation and intervention activities.

Additionally, various frameworks have been developed to facilitate the implementation planning process, and this study sought to utilize the RE-AIM and the CFIR frameworks for quality improvement within EDs to reduce wait times. This combination provides the researchers the opportunity to capture and address a “taxonomy of contextual” factors and processes (e.g., inner and outer setting) behind the results of five key dimensions of an intervention to facilitate data collection and analysis [20, 37].

Furthermore, using the variety of quantitative and qualitative methods allows us to comprehensively address all RE-AIM dimensions and compare factors that are essential to the success or failure of the implementation that otherwise could not feasibly be captured. Employing qualitative methods also provides various opportunities for the participants to fully engage in all stages of implementation and evaluation of the intervention. This leads to a team-based approach that not only involves the multi-disciplinary team and relevant stakeholders (e.g., nurses, physicians, administers, etc.) in planning, implementing, and evaluating the intervention program, but also patients, who are provided with different opportunities (e.g., surveys, interviews, etc.) to engage with the team. Additionally, by involving four different hospitals of varying sizes and locations, this program aims to gain as much rich information as possible concerning the implementation challenges across different contexts.

Finally, one of the goals of the SurgeCon project are knowledge translation and transforming ED practice into an improved, more efficient system. Our partnership with the largest health authority in our province (EH) provides opportunities in both rural and urban ED environments for implementing our findings^2^.

## Data Availability

Not applicable.

## Abbreviations

ED: Emergency department
RE-AIM: Reach, Effectiveness, Adoption, Implementation, and Maintenance
CFIR: Consolidated Framework for Implementation Research
NL: Newfoundland and Labrador
ICT: Innovative Clinical Trial
GT: Grounded Theory
EH: Eastern Health
RHA: Regional Health Authority
PIA: Physician’s initial assessment
KPI: Key performance indicator
NLCHI: Newfoundland and Labrador Centre for Health Information
GLMM: Generalized linear mixed modeling

## Footnotes

1 The Carbonear ED was excluded since it was the pilot site of the intervention, and the Janeway Children’s hospital ED was excluded since it only provides care to children and adolescents and did not have excessive wait times.

2 This study adheres to the SPIRIT 2013 reporting guideline [38].
